# Supplementary figures and images for: Single-cell alternative polyadenylation analysis reveals mechanistic insights of COVID-19-associated neurological and psychiatric effects
Source: PLoS One. 2025 Dec 26;20(12):e0324689. doi: 10.1371/journal.pone.0324689 (PMC12742782; doi:10.1371/journal.pone.0324689)

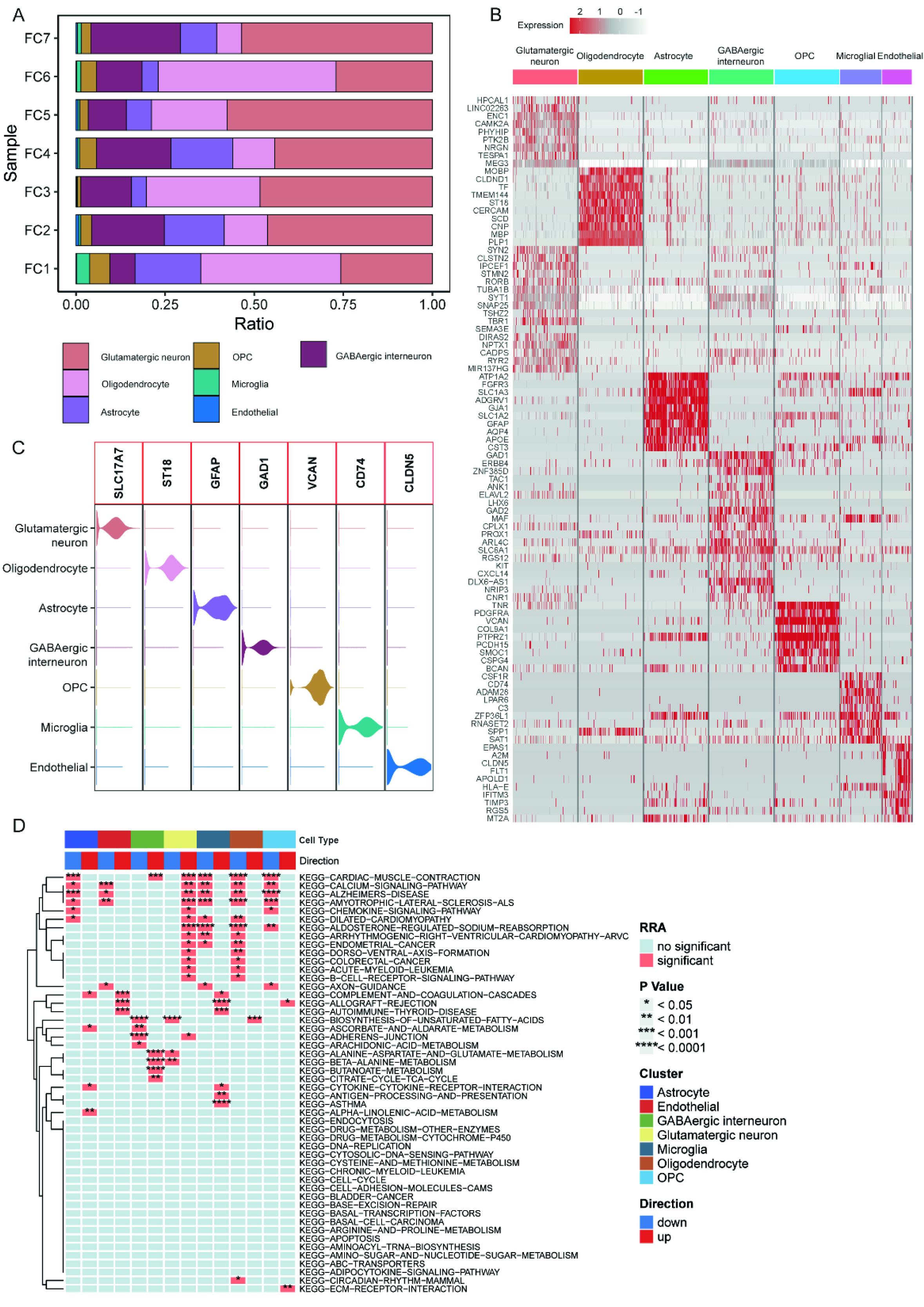

Supplement: S1 Fig — (A) Each cell type proportion in 7 control individuals. (B) Heatmap plot showing the top 10 most differentially upregulated genes in each cell type identified through unsupervised clustering. Red indicates higher expression; Grey indicates lower expression; the Average expression (avg. exp) scale is shown on the top. (C) Violin plot showing the expression level of cell markers with significantly different percentages per cell type. (D) Heatmap plot showing co-upregulated or co-downregulated gene sets per cluster in RRA. Red indicates up-regulated; Blue indicates down-regulated. (TIF) [file pone.0324689.s001.tif]

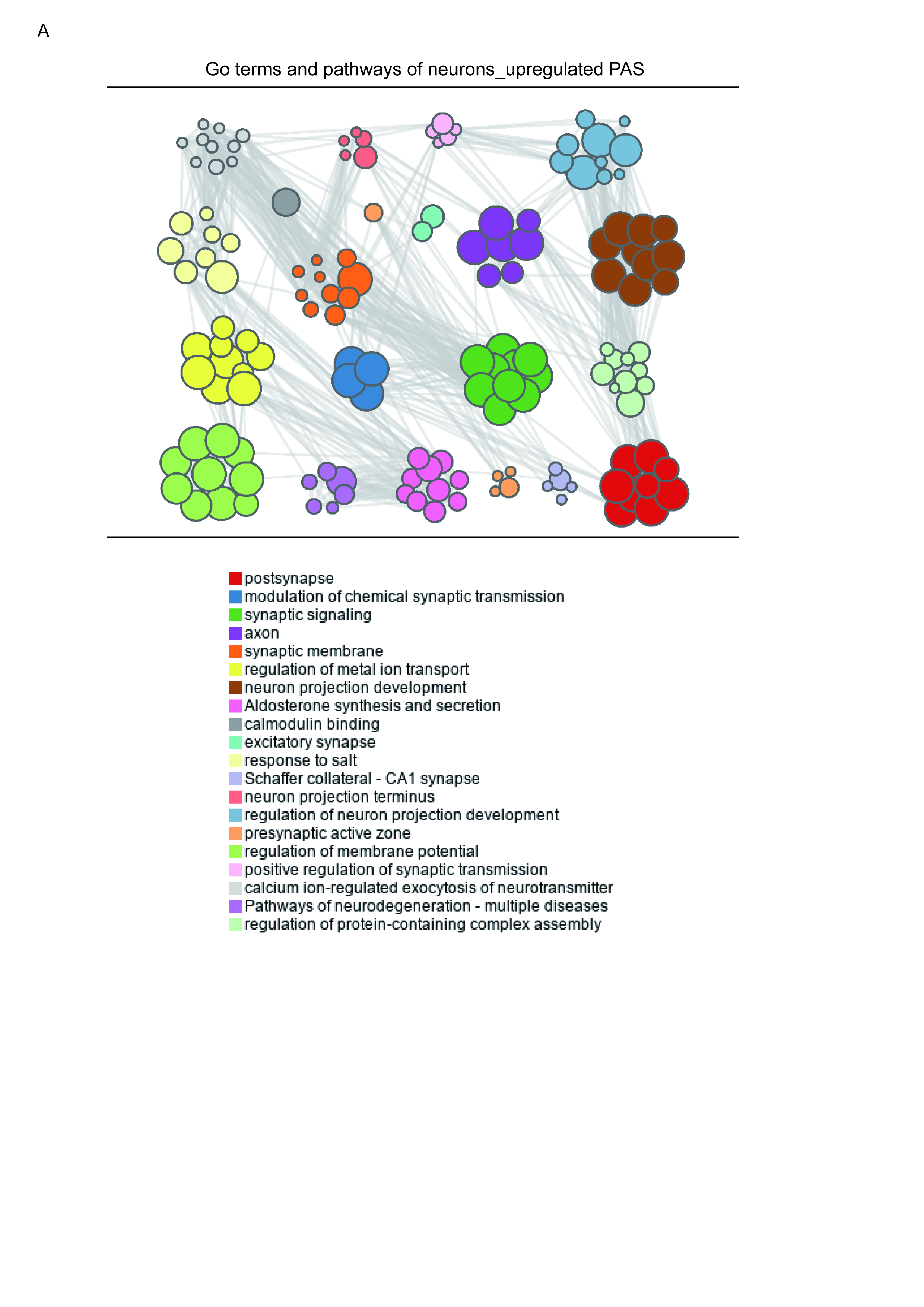

Supplement: S2 Fig — GO terms and pathways enriched among genes with differential PAS usage in neurons compared to non-neuronal cells. The size of each network node represents the number of genes associated with the corresponding term. Terms with a similarity score > 0.3 are connected by edges. (TIF) [file pone.0324689.s002.tif]

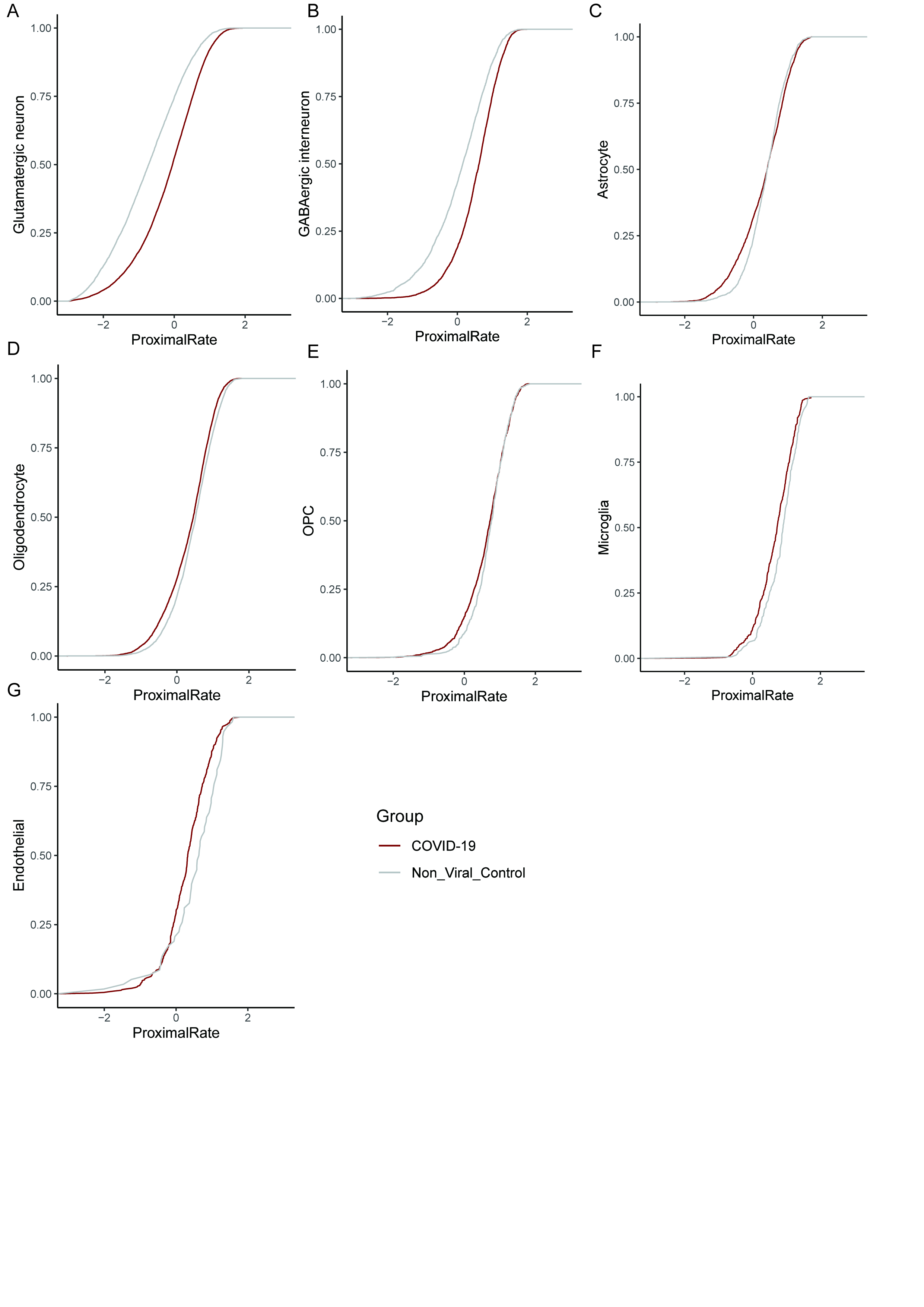

Supplement: S3 Fig — Scaled proximal usage index (PUI) values (x-axis, ranging from –3–3) are plotted for different cell types, with values representing normalized expression levels. The y-axis shows the accumulation rate. Grey lines indicate the accumulation rate for each cell type in the non-viral control group, and red lines indicate the COVID-19 group. Statistical significance was assessed by Kolmogorov-Smirnov test, and p values are indicated for each cell type: (A) glutamatergic neurons (p < 2.2e-16). (B) GABAergic interneurons (p < 2.2e-16). (C) oligodendrocyte (p = 1.282e-9). (D) OPC (p = 1.623e-2). (E) astrocytes (p = 1.238e-8). and (F) microglia (p = 1.293e-3). (TIF) [file pone.0324689.s003.tif]

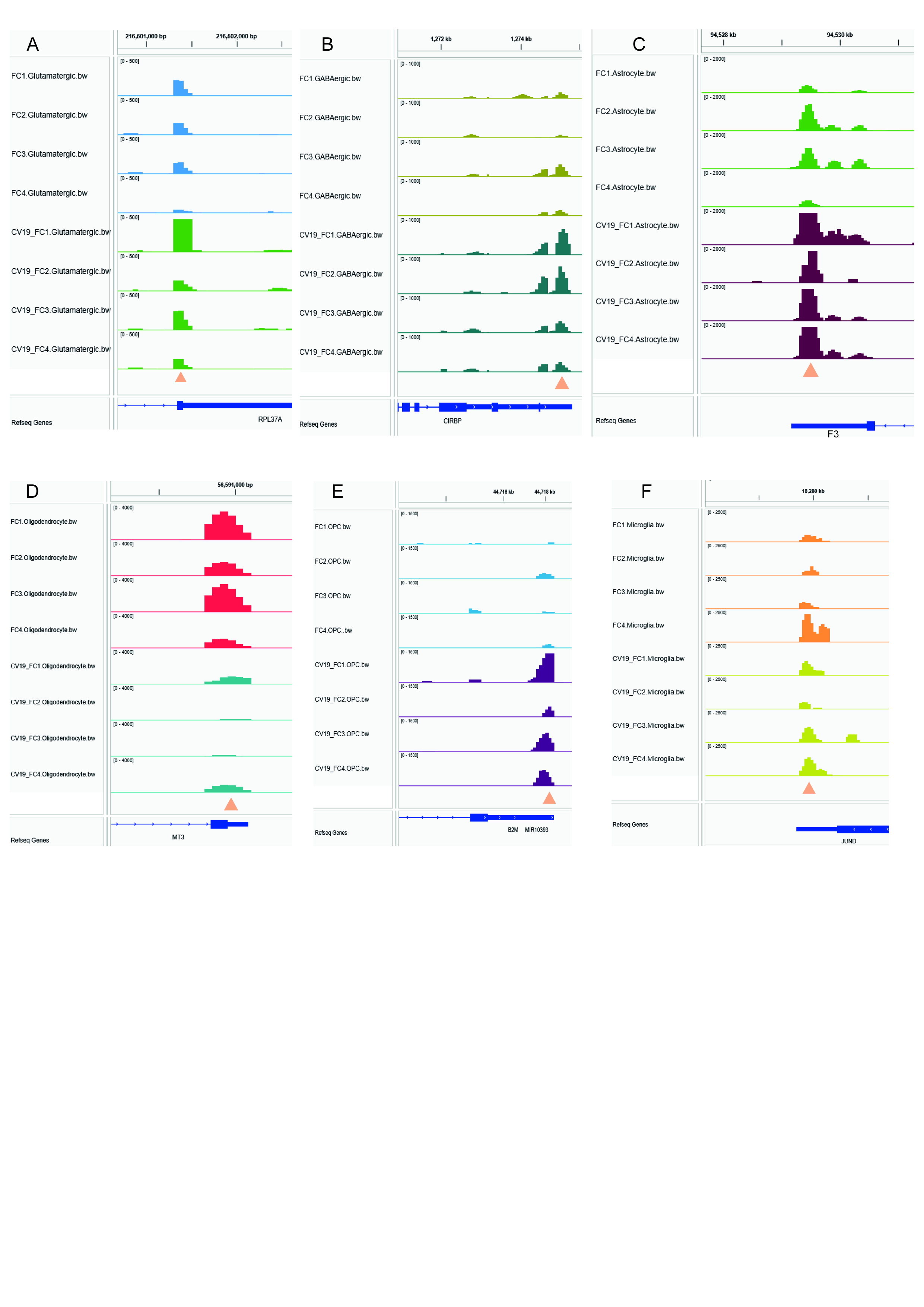

Supplement: S4 Fig — (A) RPL37A gene in glutamatergic neurons. (B) CIRBP gene in GABAergic interneurons. (C) F3 gene in astrocytes. (D) MT3 gene in oligodendrocytes. (E) B2M gene in OPC. (F) JUND gene in microglial. The same color means that the samples belong to the same group; all the tracks keep the same data range, and the triangle at the bottom indicates the PAS sites. (TIF) [file pone.0324689.s004.tif]

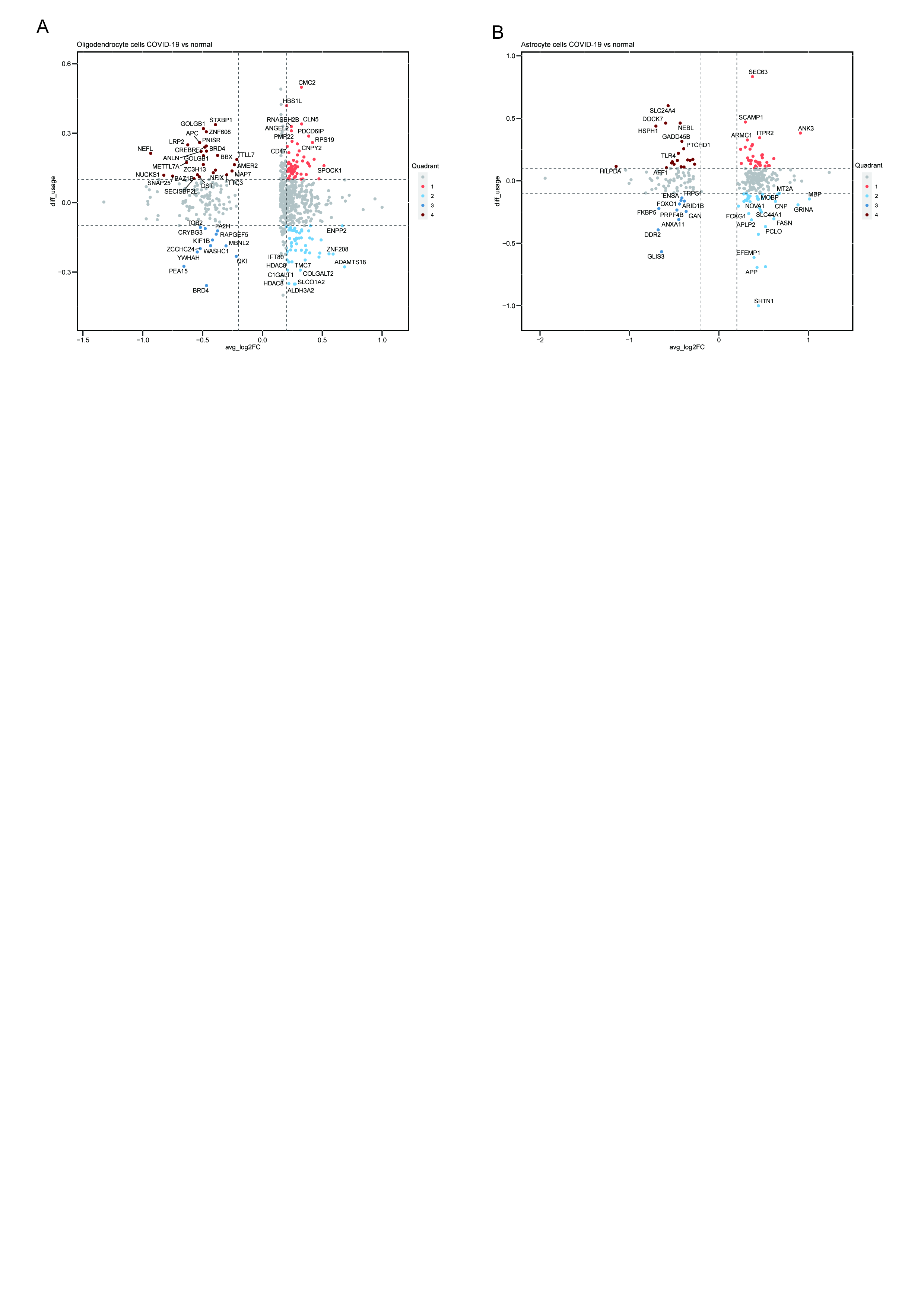

Supplement: S5 Fig — (TIF) [file pone.0324689.s005.tif]

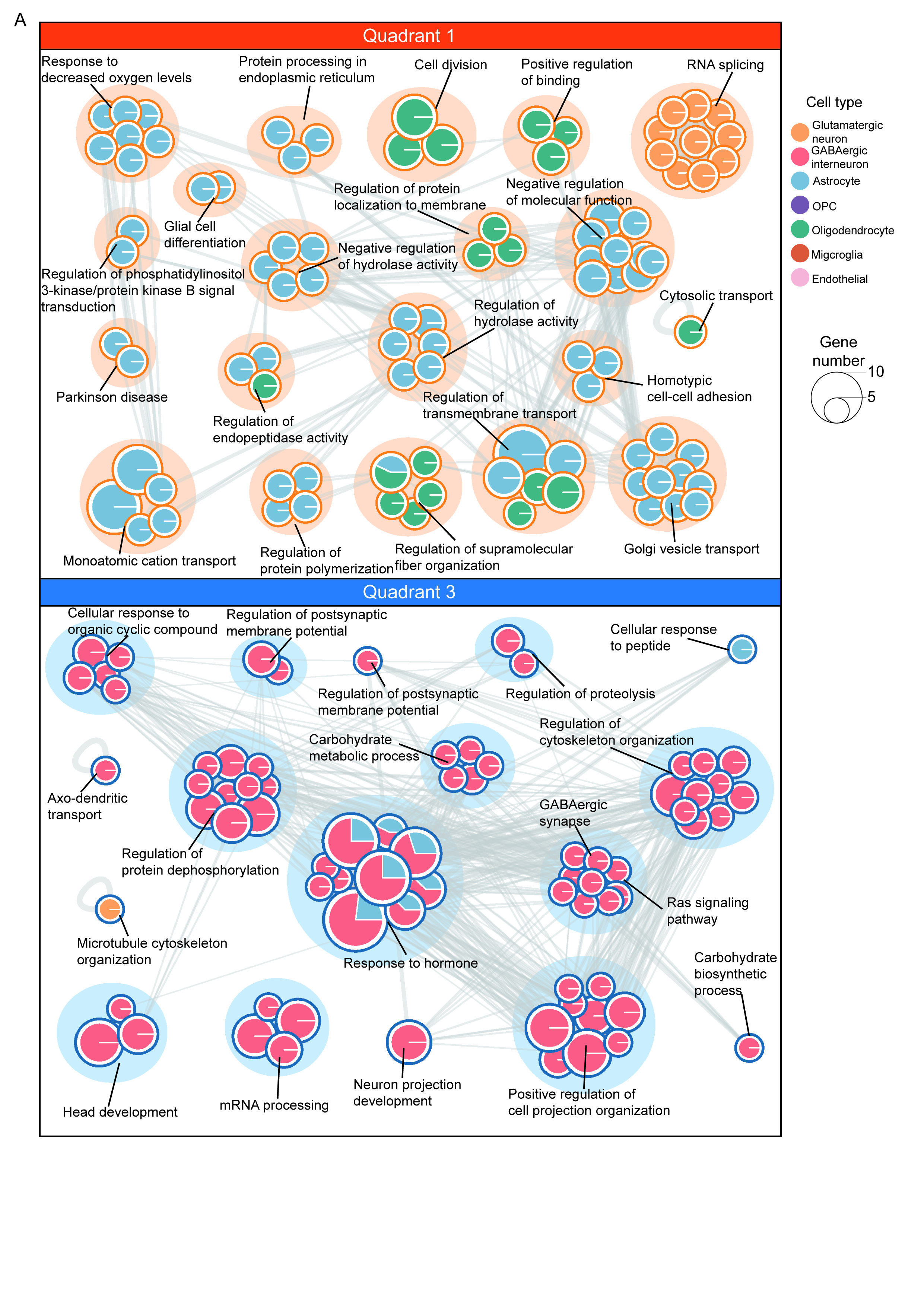

Supplement: S6 Fig — (A) The network nodes were displayed as pies. Each pie sector is proportional to the number of hits originating from a gene list. The pie charts are colored by cell types, where the size of a slice represents the percentage of genes under the term that originated from the corresponding cell type. Terms with a similarity score > 0.3 are linked by an edge (the thickness of the edge represents the similarity score). (TIF) [file pone.0324689.s006.tif]

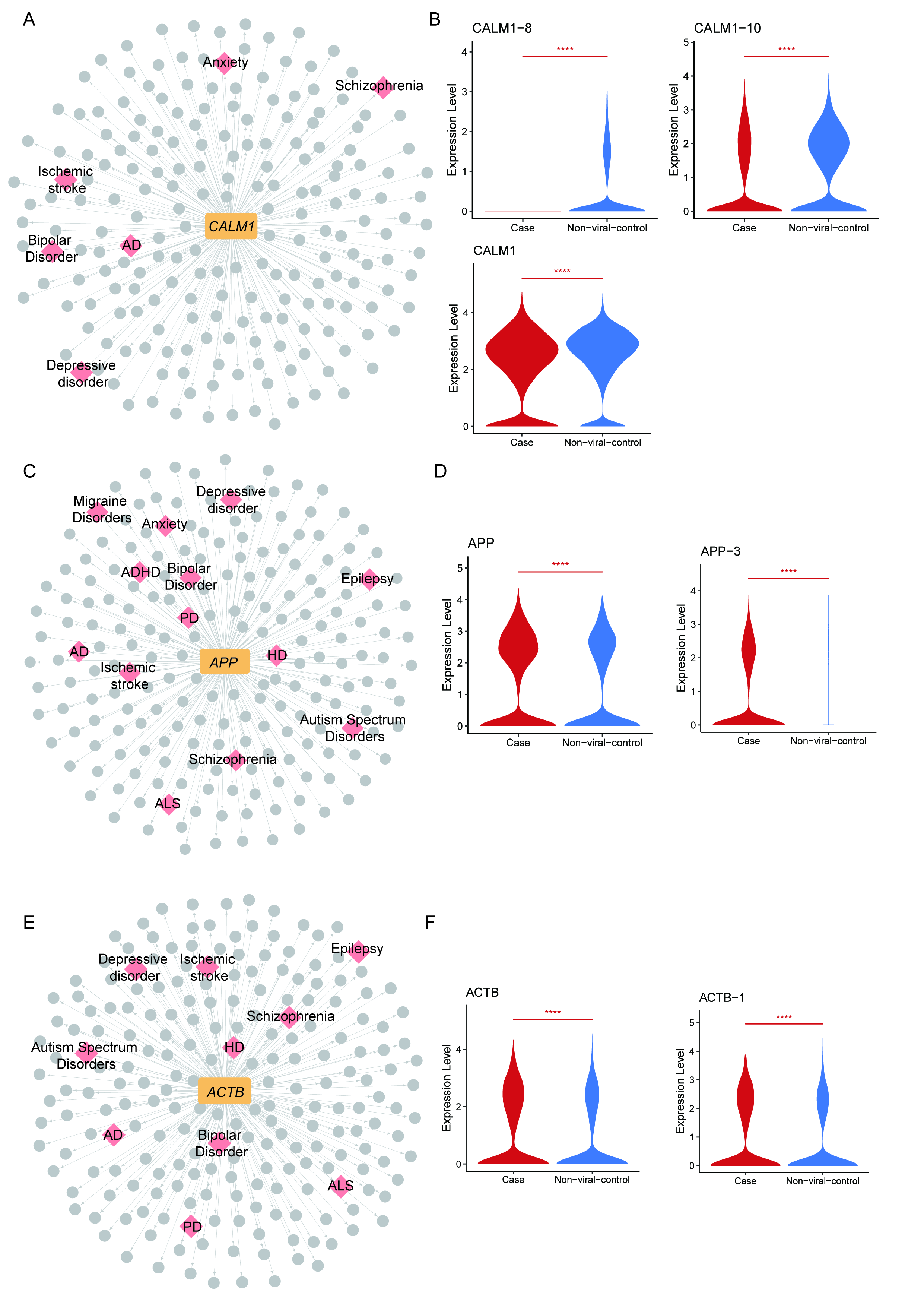

Supplement: S7 Fig — The colors of grey, blue, and orange nodes represent targeting miRNAs, genes, and diseases, respectively. Relative gene expression and APA levels of (B) CALM1 in glutamatergic neurons, (D) APP in astrocytes, and (F) ACTB in astrocytes, comparing the control group to the COVID-19 group. (TIF) [file pone.0324689.s007.tif]
