# Supplementary material for: Single-cell alternative polyadenylation analysis reveals mechanistic insights of COVID-19-associated neurological and psychiatric effects
Source: PLoS One. 2025 Dec 26;20(12):e0324689. doi: 10.1371/journal.pone.0324689 (PMC12742782; doi:10.1371/journal.pone.0324689)
Supplement: Attachment — Submitted filename: Comments to author.pdf [file pone.0324689.s018.pdf]

Chen *et al*. presents a precise and biologically pertinent exploration of alternative polyadenylation (APA) landscapes in neural cells, both under homeostatic conditions and following SARS-CoV-2 infection. Leveraging SCAPTURE strengthens data credibility, while cell-type and infection-driven APA dynamics offer valuable insights. The author identified that alternative polyadenylation patterns—specifically poly(A) site (PAS) choice—vary between cell types in the brain. Moreover, these APA events significantly alter microRNA-binding landscapes in brain cells by changing 3′ UTR lengths, which in turn affects miRNA regulation.

Major comments

1) **Figures 2A and 2C**: The visual differences between groups are subtle and not readily apparent by eye. In Figure 2A, the zoomed-in view may exaggerate the difference. Please provide the exact numerical differences and clarify whether the observed changes are statistically significant.
2) **Figure 3A**: To improve clarity, consider labeling the proximal and distal APA events directly within their corresponding quadrants. This would make the figure easier to interpret.
3) **Cell Type Proportions**: Including explicit percentages or cell counts for major populations (e.g., "glutamatergic neurons comprise X %") in the main text—not solely in Supplementary Figure 1A—would enhance the clarity and accessibility of the results.
4) **Figure 3F**: The visual differences are not apparent by eye. Please include the exact values or fold-changes to better support the interpretation.
5) **Sample Metadata and Quality Metrics**: For transparency and reproducibility, include key donor/sample metadata (e.g., age, sex, health status), along with technical metrics such as UMI counts per nucleus, read depth, and mapping quality. This will help validate consistency across the seven samples.
6) **Statistical Analysis**: Clearly specify the statistical tests used (e.g., Kolmogorov–Smirnov test) and the thresholds for significance when comparing APA shifts between infected and control groups.
