# Supplementary material for: Single-cell alternative polyadenylation analysis reveals mechanistic insights of COVID-19-associated neurological and psychiatric effects
Source: PLoS One. 2025 Dec 26;20(12):e0324689. doi: 10.1371/journal.pone.0324689 (PMC12742782; doi:10.1371/journal.pone.0324689)
Supplement: Attachment — Submitted filename: 1Comments to author.pdf [file pone.0324689.s020.pdf]

The authors have thoroughly addressed the major concerns raised during the previous review. The revised manuscript shows improved clarity, methodological transparency, and appropriate caution in interpreting qualitative data. The only remaining recommendation is to consider adding a compact summary of technical metrics (e.g., UMI counts, sequencing depth, mapping quality) across samples to further support reproducibility.

Once this minor point is addressed, I believe the manuscript will be suitable for publication.
