# Supplementary material for: Single-cell alternative polyadenylation analysis reveals mechanistic insights of COVID-19-associated neurological and psychiatric effects
Source: PLoS One. 2025 Dec 26;20(12):e0324689. doi: 10.1371/journal.pone.0324689 (PMC12742782; doi:10.1371/journal.pone.0324689)
Supplement: Attachment — Submitted filename: Point by point response.docx [file pone.0324689.s021.docx]

Dear Editor,

Thank you for the decision letter, informing us that our manuscript is potentially acceptable for publication in *PLOS One*. We would like to thank reviewers and editor for their helpful comments and suggestions. We have carefully addressed their concerns as outlined in the attached file. Point-by-point responses are followed by this cover letter. We hope that we have adequately addressed all reviewers’ and editor’s concerns and have made this revised manuscript acceptable for publication in *PLOS One.*

Sincerely,

Wanshan Ning, PhD

Professor of Bioinformatics,

Institute for Clinical Medical Research

the First Affiliated Hospital of Xiamen University,

Xiamen University

55 Zhenhai Road, Xiamen, Fujian, China 361003

E-mail: ningwanshan@xmu.edu.cn

**Responses to the editor and reviewers**

We thank the editor and reviewer for their thorough assessment and thoughtful suggestions, which helped improve this manuscript. We have incorporated the suggestions in the revised version of this manuscript, and a point-by-point response to the reviewer's comments is presented below.

**Reviewer #1:**

Major Comments

1. APA vs Neurological Symptoms Correlation

The manuscript would be strengthened by explicitly correlating the observed APA/PAS changes in each COVID-19 sample with the neurological symptoms of each corresponding patient. For instance, the supplementary data from Yang et al. (2021, Wyss-Coray Lab) includes patient-level neurological assessments that could be referenced.

Response: We thank you for the suggestion to correlate APA/PAS changes with patient-level neurological symptoms. The neurological symptom data provided in the original publication by Yang et al. (Nature, 2021, 595:565–571) are limited to brief qualitative descriptions (e.g., “1× generalized tonic-clonic seizure”, “prolonged psychosyndrome, ischemic brain lesions”, or “none reported”) for eight COVID-19 donors. These records lack standardized severity scoring, duration, or quantitative measurements, and the small number of cases with heterogeneous symptom types precludes robust statistical correlation analyses with APA/PAS changes. To address your interest, we have added a supplementary table (Supplementary Table 10.6) summarizing the reported neurological symptoms alongside our sample-level APA/PAS changes, allowing qualitative inspection of potential patterns. This comparison is intended for descriptive purposes only and no formal statistical inference is made. The supplementary table is referenced in the Results section (Line 320).

2. Lack of Evidence for Neuronal Cell Death

The manuscript implies functional impairment in neurons after infection, but does not provide direct evidence of neuronal death. Are neurons dying, or merely losing their molecular identity by downregulating distal PAS usage typical of differentiated neurons? This distinction should be clarified with appropriate data or cautious interpretation. Proximal PAS and shorter 3’UTRs are present in stem cells and dividing cells, for example. (Reference: Sommerkamp P, Cabezas-Wallscheid N, Trumpp A. Alternative Polyadenylation in Stem Cell Self-Renewal and Differentiation. Trends Mol Med. 2021 Jul;27(7):660-672. doi: 10.1016/j.molmed.2021.04.006. Epub 2021 May 11. PMID: 33985920.)

Response: We appreciate your careful reading and note that our manuscript does not state or imply direct evidence of neuronal death. Our conclusions are limited to transcriptomic changes consistent with altered APA patterns after SARS-CoV-2 infection. We recognize, however, that the observed increase in proximal PAS usage and shorter 3’UTRs could be interpreted in different biological contexts. As highlighted by Sommerkamp et al. (Trends Mol Med, 2021), shorter 3’UTRs are characteristic of neural stem/progenitor cells and other proliferative states, whereas differentiated neurons tend to express longer 3’UTRs. To avoid potential misinterpretation, we have revised the text to explicitly note that these changes may reflect a shift in molecular identity or stress response, rather than neuronal death at lines 580-585.

3. Mechanistic Insight Lacking

The study does not clearly elucidate the mechanism by which SARS-CoV-2 infection leads to the observed changes in APA and PAS site usage. This weakens the causal link between viral infection and APA dysregulation. Further experimental validation (or at least deeper discussion of known molecular mechanisms) would be helpful.

Response: We appreciate your suggestion to elaborate on potential mechanisms linking SARS-CoV-2 infection to the observed APA/PAS alterations. While our study is observational in nature and does not experimentally dissect causal pathways, previous studies have shown that viral infections can modulate APA through multiple molecular processes, including changes in the expression or activity of core 3’-end processing factors, RNA-binding proteins, and transcriptional elongation dynamics. For example, innate immune activation can alter the abundance and phosphorylation status of cleavage and polyadenylation specificity factors (CPSF) and cleavage stimulation factors (CstF), thereby favoring proximal PAS usage. Inflammatory cytokines and interferon responses have also been reported to reprogram RNA processing machinery in infected cells. Moreover, coronaviruses, including SARS-CoV-2, can directly or indirectly influence host transcription termination and RNA stability, which may contribute to the APA landscape changes we observe. We have incorporated these considerations into the revised Discussion to provide a more complete mechanistic context for our findings.

4. Focus on Neurons vs Astrocytes

The authors report that astrocytes exhibit more unique upregulated PAS events than neurons. Why then is the primary focus placed on neurons? A rationale for this choice should be discussed.

Response: We appreciate your observation regarding the relative abundance of upregulated PAS events in astrocytes compared to neurons. Our focus on neurons was based on several considerations. First, in the control group, neurons-both glutamatergic and GABAergic-display a lower average proximal usage index (PUI) than other brain cell types, consistent with their preferential use of distal PAS and consequently longer 3’UTRs under physiological conditions, as previously reported. This APA pattern is tightly linked to neuronal identity, maturation, and specialized functions such as synaptic plasticity. Therefore, the infection-associated shift toward increased proximal PAS usage in neurons represents a marked deviation from their baseline state and may have important functional consequences. Second, many of the APA-regulated genes identified in neurons are implicated in synaptic function, cognition, and neurodegenerative disease pathways, which are directly relevant to the neurological and psychiatric symptoms reported in COVID-19 patients. In contrast, while astrocytes show more unique upregulated PAS events in absolute number, the baseline APA profile of astrocytes is already enriched for shorter 3’UTRs, and the observed changes after infection may reflect a less dramatic departure from their baseline APA state. Taken together, we prioritized neurons for in-depth analysis because their infection-induced APA changes represent a substantial shift from their normal transcriptomic configuration and have potentially greater relevance to the functional impairments observed in COVID-19 associated brain pathology.

5. Interpretation of NEFL Data

The NEFL gene is highlighted as showing a switch to distal PAS, yet this is clearly demonstrated in only one of four samples. The evidence should be presented more cautiously, acknowledging variability.

Response: We thank you for the critical evaluation of NEFL data. We acknowledge that the distal PAS usage shift in NEFL is most pronounced in one infected sample (CV19-FC3), with more modest or inconsistent trends across others. To address this variability, we have revised our description of NEFL results to reflect that the APA change is suggestive but not universally consistent. Accordingly, we now describe this observation as “a sample-specific trend toward distal PAS usage in NEFL” and caution against overgeneralizing this finding.

6. APP 3’UTR Interpretation

In the Discussion (line 532), the claim that longer 3’UTRs increase APP translation needs more nuance. In wild-type contexts, longer 3’UTRs can enhance or inhibit translation depending on context. The problem in AD arises with mutant APP aggregation, not merely expression changes of the wild-type gene. Please clarify.

Response: We thank you for pointing out the need for a more nuanced discussion of APP 3’UTR regulation. We agree that the impact of 3’UTR length on translation efficiency is context-dependent and can either enhance or inhibit protein synthesis depending on the presence of specific RNA-binding proteins, microRNA target sites, and secondary structures. The report by Mbella et al. demonstrated that in their experimental system, the longer APP 3’UTR enhanced translation relative to the shorter form, but this is not a universal rule. In addition, we agree that Alzheimer’s disease (AD) pathogenesis is primarily associated with aberrant aggregation of mutant or misprocessed APP and Aβ peptides, rather than expression changes of the wild-type gene alone. In our discussion, the reference to altered APP 3’UTR length was intended to illustrate that dysregulated APA could influence APP expression and potentially modify the cellular environment in which pathogenic APP processing occurs. We have revised the text to clarify that (i) the translation effect of 3’UTR length is context-specific, and (ii) altered expression of APP due to APA may modulate, but is not, by itself, sufficient to cause, AD pathology.

Minor Comments

• The writing throughout the manuscript contains multiple grammatical and syntactical issues that impact clarity. Specific problematic lines include (but are not limited to): 76, 80, 105-107, 187, 192, 219, 254, 271-273, 298, 300-301, 393, 451, 501, and 562. A thorough English language edit is necessary.

Response: We appreciate you for pointing this out. We have thoroughly revised the manuscript for grammatical and syntactical issues. In particular, we have carefully addressed the sentences at lines 75, 79, 104-107, 224, 229, 260, 302, 326-330, 354, 356-358, 457, 519, 576, and so on, as pointed out. All changes have been marked in the revised manuscript.

• The sentence in lines 341-343 should be revised for clarity. Suggested rewrite:

“We calculated APA usage by subtracting the usage value in the control group from the infected group. We considered changes with an absolute value greater than 0.1 to be significant.”

Response: Thank you for the suggestion. We have revised the sentence in lines 396-398 for clarity as suggested.

• A reference should be added for the movAPA software used in the analysis.

Response: Thank you for your comment. The reference for movAPA has already been included in the manuscript as Reference [17]. We have now double-checked and made sure it is clearly cited when the software is first mentioned in the Materials and Methods section.

• Consider updating the running title to:

“Effects of COVID-19 on single-cell alternative polyadenylation”

for better clarity and relevance.

Response: Thank you for the helpful suggestion. We have updated the running title to: “Effects of COVID-19 on single-cell alternative polyadenylation” to improve clarity and better reflect the focus of the study.

• The reviewer was unable to access some supplementary tables due to possible malware alerts. Ensure all supplementary materials are securely hosted and accessible.

Response: We thank you for pointing this out. All supplementary tables were generated as standard .xlsx files and can be opened without any alerts on our local systems. We have re-checked each file and confirmed that they contain no macros or embedded content that could trigger security warnings. To ensure accessibility, we have re-exported the tables from the original data and re-uploaded them to the journal submission system as fresh, clean files.

• Supplementary Figure 1: The proportion of neurons varies dramatically among the 7 control individuals. If these samples were taken from similar brain regions, such inter-individual variability should be minimal. Could the authors comment on potential batch effects or anatomical heterogeneity?

Response: We thank you for highlighting the inter-individual variation in cell-type composition. As shown in Supplementary Figure 1A, glutamatergic neurons constitute the largest population in most control samples, followed by oligodendrocytes and GABAergic interneurons, while endothelial cells and microglia remain relatively rare. We note that in two control samples (FC1 and FC6), the proportion of neurons is lower and oligodendrocytes are more prevalent compared with the other individuals. This discrepancy may reflect a combination of biological heterogeneity (e.g., subtle anatomical differences within the frontal cortex sampling sites) and technical variation. To minimize the influence of such variability, we applied the Harmony integration algorithm in downstream analyses, as detailed in the Methods section.

• Supplementary Figure 2: The figure lacks adequate explanation. Specifically, it is unclear what the circle sizes represent. Please clarify this in the figure legend and/or main text.

Response: Thank you for the helpful comment. We have revised the legend of Supplementary Figure 2 to clarify the meaning of the circle sizes. Specifically, we now state that the size of each node represents the number of genes associated with the corresponding GO term or pathway. This clarification has been added to the figure legend.

• Supplementary Figure 3: The y-axis label is missing or unclear. What parameter is being plotted (e.g., Proximal Usage Index, expression level, etc.)? This should be clearly defined.

Response: Thank you for pointing this out. In Supplementary Figure 3, the proximal usage index (PUI) is plotted on the x-axis, with values scaled from -3 to 3. The y-axis represents the accumulation rate for each cell type. We have revised the figure legend to clarify this information. In the plot, grey lines represent the accumulation rate of each cell type in the non-viral control group, while red lines represent the COVID-19 group.

• Supplementary Figure 7: It is unclear whether the data presented reflect all cell types combined or specific cell subtypes. Please indicate which cell populations are represented in the plots and whether differences exist across subtypes.

Response: Thank you for the valuable comment. As described in the manuscript (Lines 514-534), Supplementary Figure 7 presents APA and expression changes of selected disease-related genes in specific cell subtypes. To improve clarity, we have now explicitly stated the involved cell populations in the figure legend and ensured that the main text highlights this more clearly.

Recommendation:

Minor Revision

While the manuscript presents valuable data, improvements in writing, clarification of biological interpretations, and cautious framing of key claims are needed to meet PLOS ONE's publication standards.

**Reviewer #2:**

Revisions (marked with +, ++, +++ or ++++ to determine their importance):

++++ In a general manner, the authors tend to be obscure in the way they are choosing, interpreting or reporting results. Also, figure legends do not seem to be complete. Here are examples that should be clarified:

+ Line 35: “some prefer short isoform” should be rephrased

Response: We thank you for the suggestion. The phrase “some prefer short isoform” has been rephrased for clarity.

+ Line 66-68: Could the authors explain, or clarify? They first say the genome is more broadly expressed in neuron, explaining the large mRNA diversity and then; they say the large mRNA diversity is due to alternative posttranscriptional mechanisms...

Response: We thank you for pointing out the potential ambiguity. Our intention was not to present two unrelated causes, but to describe a sequential relationship: neurons and peripheral nerve tissues express a larger fraction of the genome compared with other tissues, which provides the basis for higher mRNA diversity. This diversity arises primarily from post-transcriptional regulatory mechanisms, such as alternative splicing, alternative transcription initiation sites, and alternative polyadenylation (APA). To avoid misunderstanding, we have revised the text.

+ Line 108: rephrase in appropriate English

Response: Thank you for the suggestion. We have revised the sentence in Line 110 to improve clarity and grammatical accuracy.

+ Line 113-114: rephrase in appropriate English

Response: We appreciate your comment. The sentence has been rephrased to enhance clarity and precision in Line 115-117. The updated sentence now reads: “After quality control, the data were further normalized, scaled, and filtered for highly variable features using Seurat functions NormalizeData, ScaleData and FindVariableFeartures prior to principal component analysis (PCA).”

++ Lines 140-146: The authors explain the PUI concept. While the metric might be appropriate, the authors do not explain appropriately how it is relevant as a metric. They also should clarify if they only used genes including only two PAS since it is what is implied by their phrasing.

Response: We thank the reviewer for the insightful comment. To improve clarity, we have revised the paragraph in Lines 152-167 to more explicitly explain the rationale for using PUI as a metric of APA preference. The PUI provides a quantitative measure of the relative preference for proximal versus distal PAS usage at the transcript level, making it suitable for capturing APA dynamics across cell types and conditions. Additionally, we clarify that only transcripts with at least two annotated PASs (one proximal and one distal) were included in the calculation of PUI. Transcripts with only a single PAS were excluded, as they do not provide sufficient information for assessing alternative polyadenylation.

+ Lines 149-151: The author should clarify their choice in the transcripts (p value or p adj? why log2 fold change larger than 0.25?)

Response: We appreciate your comment. In our analysis, we used adjusted p-values (p_val_adj) to control for false discovery rate (FDR) from multiple testing, with a threshold of p_val_adj < 0.05. Regarding the log₂ fold change threshold, we set |avg_log2FC| > 0.25 because in single-cell alternative polyadenylation (APA) analyses, the magnitude of changes is generally smaller than in gene expression analyses. A lower threshold allows us to capture biologically relevant APA shifts while minimizing the exclusion of meaningful events.

+ Line 174: “poly(A)” is a confusing term in that case because it refers to polyadenylation while it usually refers to polyadenosine stretches. It should be changed for clarity.

Response: Thank you for pointing this out. We agree that the term “poly(A)” may cause confusion, as it is commonly associated with polyadenosine sequences. To improve clarity, we have revised the sentence in Line 206 to use “polyadenylation (poly(A)) sites” upon first mention and clarified the genomic context in which these sites were identified.

+++ Figure 1G - Lines 216-220: The authors show that some cell types use more diverse PAS than others. However, the population showing the highest diversity are also the most abundant. This abundance increases the probability of detecting low abundance APA. The authors should use cell subsets of the same size to determine the APA abundance instead of using the entire cell subpopulations.

Response: Thank you for raising this important point. In our analysis, PAS detection and diversity were calculated based on normalized expression values per cell type, and the final metrics were obtained after aggregating by mean expression, rather than directly counting individual cells. While we acknowledge that larger cell populations have a higher probability of detecting low-abundance PAS, the main conclusions we report (e.g., higher PAS diversity in neurons) are consistent with the biological characteristics of these cell types and are unlikely to be driven solely by differences in cell numbers. We have clarified this point in the revised manuscript.

+ Lines 244-245: The legend should be completed for the readers to know what are the values behind each box of the boxplot (interquartile range? 5%-95%? Min-Max?)

Response: We appreciate the reviewer’s helpful comment. The boxplots were generated using standard statistical settings, where the boxes represent the interquartile range (IQR: 25th–75th percentile), the horizontal line within the box indicates the median, and the whiskers extend to 1.5×IQR from the quartiles. We have now clarified this information in the corresponding figure legend.

+ Line 252-255: Can the authors comment on the decrease in the oligodendrocyte population from non-infected to infected?

Response: We thank the reviewer for this comment. In our snRNA-seq dataset, we observed a lower proportion of oligodendrocytes in the COVID-19 group compared to the non-viral control group. Oligodendrocytes play a critical role in the central nervous system (CNS) by wrapping axons, forming insulating myelin sheaths, facilitating rapid saltatory conduction of electrical impulses, and maintaining and protecting normal neuronal function. Dysfunction of oligodendrocytes can lead to CNS demyelinating diseases, neuronal injury, or psychiatric disorders. This reduction in oligodendrocytes may reflect SARS-CoV-2–related neuropathology, as previous studies have reported oligodendrocyte loss or myelin dysregulation in response to infection and neuroinflammatory processes. Notably, “Mild respiratory COVID can cause multi-lineage neural cell and myelin dysregulation” reported that even mild respiratory SARS-CoV-2 infection can lead to persistent loss of oligodendrocytes and myelinated axons.

Reference: Fernández-Castañeda A, Lu P, Geraghty AC, et al. Mild respiratory COVID can cause multi-lineage neural cell and myelin dysregulation. *Cell*. 2022 Jul 7;185(14):2452-2468.e16. doi: 10.1016/j.cell.2022.06.008.

+++ Line 261: “relatively high significant preference” --> no mention of the statistics are made anywhere + “preference” is not appropriate + the sentence is hard to read

Response: We thank you for pointing this out. We have revised the sentence to improve clarity, replace the term “preference” with a more precise description, and explicitly mention the statistical test used. In the revised version, we state that we assessed the significance of changes using the Kolmogorov-Smirnov test and describe the results as a “shift toward proximal PAS usage” rather than a “preference”.

++++ Lines 266 - 276 + Figure 2E-F: The Sankey plot is inappropriate and unintelligible. The authors should find a more appropriate way to display the data. Same for Figure 2F that doesn’t display a ratio as the authors suggest. Also, a binary red/blue scale is not appropriate to show “increase/decrease”. My suggestion would be one slope chart per cell type (x axis = Distal/Proximal; y axis = number of reads (CPM)). I didn’t get the impact of knowing the PAS subtype in this paragraph.

Response: We thank you for the constructive comments regarding Figures 2E-F and the associated paragraph. Our goal in using the Sankey plot (Figure 2E) was to provide a global overview of how PAS genomic location, PAS type, and cell type are connected in a single visualization. We acknowledge, however, that this format can appear complex. To improve clarity, we revised the legend and text to explicitly describe the purpose of each column and the biological message conveyed, namely that 3’UTR PAS dominate across cell types and that distal PAS usage is generally more prevalent than proximal PAS. For Figure 2F, we agree with you that showing only counts of significant PAS events may be misleading and does not reflect a “ratio” as originally stated. To address this, we normalized by calculating, for each cell type, the proportion of significant PAS (number of significant PAS/number of testable PAS, separately for up- and down-regulated events). We then compared these proportions across cell types using contingency-table tests. These results are now provided in Supplementary Table 10.2. Since these analyses confirmed the same biological trends (i.e., neurons exhibiting more downregulated PAS, whereas astrocytes and oligodendrocyte lineage cells showing more upregulated PAS), we retained the bar plot in Figure 2F for visual simplicity, while clarifying in the legend that it shows counts of significant PAS, and directing readers to the normalized proportions and statistical comparisons in Supplementary Table 10.2. We fully appreciate the reviewer’s suggestion of slope charts, but after exploring this option we found that slope charts per cell type did not scale well to the number of events and cell types in our dataset, and were less effective in conveying the global distribution patterns. We therefore opted to keep the Sankey/bar plot combination, but with clarified text and supplementary normalization, which we believe now present the data more clearly and rigorously.

+ Line 279: “in pink” doesn't relate to anything I could find easily

Response: We thank the reviewer for noting this discrepancy. The original text mistakenly referred to “pink”, whereas the correct color in the figure is “red”. We have revised the text accordingly to ensure consistency between the description and the figure.

+ Lines 292-293: “Here are the validation results of some PAS in supplementary Figure 4” is a strange way of reporting a result and should be rephrased

Response: We appreciate your suggestion. We have rephrased this sentence to improve clarity and to describe the validation process more formally.

++ Line 293: Please clarify how you chose the different PAS expressions to display

Response: We thank you for pointing out the need for clarification. In Supplementary Figure 4, our intention was to provide illustrative validation of the SCAPTURE results using IGV. To this end, we selected one representative gene from each major cell type, where differential PAS usage between the infected and control groups was most evident. The genes shown (e.g., RPL37A in glutamatergic neurons, CIRBP in GABAergic interneurons, F3 in astrocytes, MT3 in oligodendrocytes, B2M in OPCs, and JUND in microglia) were chosen to highlight clear examples of proximal vs. distal PAS shifts. The first four samples from each group were displayed, and peak heights in the IGV tracks were used to reflect relative PAS expression levels. Thus, these examples serve as representative visual confirmations across cell types, complementing the genome-wide statistical analysis rather than constituting an exhaustive list.

+ Line 298: “Take oligodendrocytes as an example” should be rephrased

Response: We appreciate your suggestion. We have rephrased this sentence to make it more formal and concise. The revised version reads: For instance, in oligodendrocytes, at the MT3 gene (Supplementary Figure 4D), the track heights in the control group are higher than those in the infected group, consistent with the results in Supplementary Table 5.

++ Line 299: To my knowledge, “the height of tracks” is not a normalized data (I might be wrong). The authors shouldn't compare non-normalized abundances.

Response: We thank you for this important point. We agree that raw track heights are not normalized and thus should not be directly compared. In our validation, however, we did not use raw bam coverage. Instead, we converted the bam files into bigwig format using deeptools, which applies per-million mapped read normalization to ensure comparability across samples. Thus, the displayed “track heights” in IGV reflect normalized coverage values rather than raw abundances. Importantly, these IGV snapshots are only used as a qualitative visualization to confirm the presence and relative location of PAS usage differences already quantified through SCAPTURE.

++ Line 305: “(|avg_logFC| > 0.25)”. Why not using p value?

Response: We thank the reviewer for the comment. We did use p-values in the selection criteria. As stated in the Materials and Methods section (Lines 170-174), transcripts were selected based on adjusted p-values (p_val_adj) < 0.05 in addition to having an average log₂ fold change greater than 0.25.

+++ Lines 342-343: “[we] define the absolute value larger than 0.1 as the significant changes of APA usage”. This seems inappropriate as an arbitrary decision shouldn't be taken as a statistically significant.

Response: We thank the reviewer for this comment. We agree that “statistical significance” should not be confused with a purely arbitrary threshold. In our analysis, the absolute usage change threshold of 0.1 was selected as a biologically meaningful cutoff based on both data characteristics and previous literature. Specifically, in our dataset, APA usage differences rarely reached large magnitudes due to the nature of single-nucleus data, so a 0.1 cutoff allowed us to capture meaningful APA changes. This approach is also supported by precedent in published work, for example in Alternative polyadenylation drives oncogenic gene expression in pancreatic ductal adenocarcinoma (Figure 5A), where a Δusage > 0.1 was used as the threshold for defining relevant APA changes. To avoid confusion, we have revised the text to clarify that this threshold is a biological effect-size criterion, and that statistical significance was evaluated separately.

Reference: Venkat S, Tisdale AA, Schwarz JR, et al. Alternative polyadenylation drives oncogenic gene expression in pancreatic ductal adenocarcinoma. *Genome Res*. 2020 Mar;30(3):347-360. doi: 10.1101/gr.257550.119.

+ Line 358: “prefer”

Response: We thank you for pointing this out. The term “prefer” is subjective and may not be precise in this scientific context. We have revised the sentence to use a more neutral and descriptive wording.

++ Line 366: UMAP seems inappropriate to determine gene expression level, authors should only keep Figure 3D as the gene expression level data. Also, statistics between both groups might be of use.

Response: We appreciate your comment. We agree that UMAP is more suitable for visualizing cell distribution rather than for quantitative determination of gene expression levels. In the revised manuscript, we have clarified that Figure 3C is intended to display the spatial distribution of NEFL expression across cell types, while Figure 3D provides the quantitative gene expression data for each cell cluster. We have retained Figure 3D as the primary source of expression level information, in accordance with the reviewer’s suggestion.

+ Line 370 - Figure 3E: the Y axis should be clarified

Response: Thank the reviewer for pointing this out. In Figure 3E, the y-axis represents the scaled proximal usage index (PUI) values, which indicate the relative usage of the proximal PAS site for NEFL-3 in each cell cluster. We have updated the figure legend to clarify this.

+ Lines 374 - 380: looks like a legend and should be rephrased

Response: We appreciate your comment and have rephrased this section to make it more suitable for the main text rather than a figure legend.

++++ Lines 388 - 411: While the authors only PREDICT miRNA-UTRs matchings, they assume “Genes with Variational APA Levels and Differential Expression Widely Affected Neural Function in MicroRNA-dependent Fashion”. They should either perform appropriate biological assays (i.e. luciferase reporter assays) or be more cautious in their conclusions. Also, the terms they identify in the functional annotation enrichment analysis are all related to normal neuron cellular life and are not surprising to be dysregulated in infected cells.

Response: We appreciate your comments and agree that our current results are based on in silico predictions of miRNA-3’-UTR interactions rather than direct biological validation. We have therefore revised the relevant section to make our conclusions more cautious and to clarify that these findings represent predictive associations rather than experimentally confirmed mechanisms.

+++ Line 442: The authors do not define what a “risk gene” is. It seems that, while they are working with transcriptomics data, they use genomics data as reporters of what is a “risk” gene. However, despite this, their examples are sound and relevant.

Response: We appreciate your comment and agree that the term “risk gene” should be defined. In our study, “risk genes” refer to genes that have been reported to be associated with specific human diseases or traits in curated genomics resources, including DisGeNET, BioKA, and the GWAS catalogue. These associations are based on prior genomic studies and do not imply direct causality in our dataset, but serve as a reference for identifying genes of potential clinical or biological relevance.

+ Line 458: “inverstAgation”

Response: Thank you for pointing out the typographical error in Line 527. We have corrected “Investagation” to “Investigation” and revised the sentence for clarity.

++++ General: the authors should try to find out which of these elements are really due to SARS-CoV-2 infection or are just due to the individuals being sick. For that, I suggest to add more controls, including (1) non-SARS-CoV-2-infected SICK patients and (2) the same patients after recovery, to avoid a selection bias.

Response: We thank you for this important suggestion. We fully agree that including additional controls, such as non-SARS-CoV-2-infected patients with other illnesses and longitudinal samples from patients after recovery, would provide stronger evidence to disentangle virus-specific effects from general sickness-related changes. Unfortunately, such samples were not available in the present dataset, which is a limitation of our current study. We have now acknowledged this point in the revised Discussion. We consider your recommendation highly valuable and will incorporate such control groups in future studies when appropriate datasets or samples become accessible.

**Reviewer #3:**

Major concerns:

Summary, Highlights, and Introduction are clear to me.

Materials and Methods

1. I have a concern about whether the input data (15 samples) chosen based on a single study (Yang et al. 2021; ref #12) is sufficient to represent the overall transcriptomics of neural cells in SARS-CoV-2-infected individuals. Taking into account the presence of a great variety across SARS-CoV-2 variants and patient variations, a detailed explanation of the data acquisition strategy that avoids any bias introduced by the selected input dataset is necessary.

Response: We thank the reviewer for this thoughtful comment. Our analyses were based on the single-cell RNA-seq dataset from Yang et al. (2021), which, at the time of our study design, represented one of the few publicly available datasets containing both SARS-CoV-2–infected and control human brain samples with sufficient sequencing depth and cell-type annotation to support APA detection. We agree that this dataset does not capture the full diversity of viral variants or patient variability, and we have explicitly acknowledged these limitations in the revised Discussion. Our study should therefore be considered exploratory, with the primary aim of highlighting potential APA regulation in neural cells during infection, rather than providing a definitive characterization across all contexts.

2. The authors mentioned “single-cell APA level” a couple of times in this manuscript; however, it is written as “sn-RNA-seq” quality control (line 104). It remains fundamentally different between scRNA-seq and snRNA-seq. Please specify what kind of dataset was used in this work.

Response: We appreciate the reviewer’s insightful comment. The dataset used in this study is based on single-nucleus RNA sequencing (snRNA-seq). The term “single-cell APA level” in our manuscript does not refer specifically to scRNA-seq, but rather to APA quantification at the resolution of individual nuclei/cells in the snRNA-seq dataset.

3. In general, the authors should provide basic information (at the minimum level) for readers to understand the analytical pipeline. A brief description (line 132) of the methodology behind data processing using SCAPTURE is recommended.

Response: We thank the reviewer for the suggestion. We have added a brief description of the SCAPTURE-based data processing pipeline in the Materials and Methods section (lines 139-150 in the revised manuscript). Specifically, we now describe that 3’ tag-based reads were processed with SCAPTURE to call polyadenylation sites (PAS), remove internal-priming artifacts via its deep-learning classifier, and generate a per-sample PAS-by-cell UMI count matrix. We also clarify that PAS identifiers and cell barcodes were harmonized across samples, lowly expressed PAS were filtered, and the resulting PAS matrices were merged into a Seurat object for normalization, integration (Harmony), dimensionality reduction, and clustering.

Results

1. Although ⅓ of the PAS were located in 3’UTRs, is this the superior proportion compared with those present in genetic features? If not, how do the authors interpret the importance of PAS that are present in 3’UTRs than other genetic features?

Response: We appreciate the reviewer’s question. In our dataset, 27.9% of PAS were located in annotated 3’UTRs, with the remainder distributed across other genic features. Although the proportion in 3’UTRs is not the majority, these sites are of particular biological interest because APA events in 3’UTRs can alter mRNA stability, localization, and translational efficiency by changing the regulatory landscape of cis-elements, such as microRNA target sites and RNA-binding protein motifs. Therefore, despite being less abundant, 3’UTR PAS have a disproportionate potential to influence post-transcriptional regulation and cellular function. We have revised the corresponding section in the Results to reflect this clarification.

2. Please debug your analysis behind Figure 1C. On the x-axis, a region named “NA” is shown. In addition, in comparison to the data shown in Figure 1A, the proportion of PAS in 3’UTRs shown in Figure 1C seems to be less than one-third compared to others. How did the authors normalize PAS counts that are summed from different genetic regions?

Response: We thank the reviewer for pointing out these issues. First, the label “NA” on the x-axis of Figure 1C was incorrectly annotated, it actually corresponds to exon PAS and has been corrected in the revised figure. Second, regarding the proportion of 3’UTR PAS: in our dataset, 27.9% of all PAS were located in annotated 3’UTRs, consistent with Figure 1A. Figure 1C, however, does not depict the total PAS distribution across genomic regions, instead, it shows the distribution of PAS associated with three specific poly(A) signal categories (“AAUAAA”, “AUUAAA”, and “Variants”), hence the proportions are not directly comparable to Figure 1A. Third, regarding the normalization of PAS counts from different genomic regions, we followed the SCAPTURE protocol, in which each PAS is annotated based on its genomic location using a hierarchical assignment. The counts shown represent the number of unique PAS sites assigned to each region, not expression-level-normalized values (e.g., CPM or UMI counts). The goal of these plots was to describe the distribution of PAS events by genomic category, rather than quantitative expression per cell. These corrections and clarifications have been made in the corresponding result, figure, and legend.

3. I have a concern about the proximal rate mean scale shown in Fig. 1D (left panel). According to Materials and Methods, the usage levels range from 0 to 1; however, the color score shown ranges from -3 to 3.

Response: We thank the reviewer for this observation. The proximal usage values indeed range from 0 to 1 in their raw form, as described in Materials and Methods. However, the color scale in Figure 1D (left panel) reflects Z-score-standardized values generated by Seurat’s default ScaleData function, which centers each feature across all cells to a mean of 0 and scales it by its standard deviation. This transformation facilitates visualization of relative variation across cell types and typically results in values within approximately -3 to 3. The raw (0-1) proximal usage values were used for all analyses and statistical tests, only the visualization was based on standardized values.

4. Line 192: Please be aware of the presence of patient variations shown in your results. Based on the rows FC1 and FC6 shown in Supplementary Fig. 1A, neuron cells are not dominant than other cell types.

Response: We thank the reviewer for pointing out the inter-patient variation in cell-type composition. As shown in Supplementary Figure 1A, glutamatergic neurons are overall the largest population across samples, followed by oligodendrocytes and GABAergic interneurons, while endothelial cells and microglia are relatively rare. We acknowledge that in certain control samples (e.g., FC6), oligodendrocytes occupy a higher proportion than neurons, reflecting biological and technical variability among individuals. This variation was addressed in downstream analyses by integrating all samples using the Harmony algorithm, as described in the Methods section.

5. In Fig. 1F, please perform statistics to verify the statistical difference between neurons and other cell types.

Response: Thank you for the valuable suggestion. We have performed statistical analysis to compare the ProximalRateMean between neurons (including glutamatergic neurons and GABAergic interneurons) and other cell types in Figure 1F. Specifically, we grouped neurons versus non-neuronal cells and applied the Wilcoxon rank-sum test to assess the significance of the difference. The results showed a statistically significant difference (p < 0.001), and the exact p-values for all pairwise comparisons have been provided in Supplementary Table 10.1. These results have been incorporated into the revised manuscript, and the figure legend has been updated accordingly.

6. Line 218: I cannot follow how the authors discriminate cell-type-specific PAS from non-cell-type-specific. No explanation can be found in Materials and Methods.

Response: We thank the reviewer for the comment and agree that the criteria for defining cell-type-specific PAS should be clarified. As described in the revised Materials and Methods, cell-type-specific PAS were identified based on their presence/absence patterns across the cell types in the control group. Briefly, for each cell type, we calculated the mean CPM-normalized PAS expression from the PAS-by-cell matrix. PAS with a mean expression > 0 in one cell type and exactly 0 in all other cell types were defined as cell-type-specific PAS for that cell type. PAS expressed (> 0) in more than one cell type were considered non-cell-type-specific. The corresponding description has been added to the Materials and Methods.

7. Line 227: Please specify the threshold used to define proximal and distal PAS and briefly mention it. Once again, I cannot find any information anywhere in this manuscript.

Response: We thank the reviewer for pointing out the missing definition. We have now added a detailed description in the Materials and Methods section (lines 152-160 in the revised manuscript). Briefly, we quantified per-cell proximal PAS usage using the Proximal Usage Index (PUI), defined as: PUI_proximal = CPM_proximal / Σ(CPM of all PAS for that gene), yielding values in [0, 1], where a higher PUI indicates preferential proximal usage. Within each gene (strand-aware), PAS were ordered by genomic position and labeled as Proximal (most 5’), Distal (most 3’), Middle (any intermediates), or Single (only one PAS). We did not apply a fixed base-pair distance threshold. For binary contrasts, “non-proximal” denotes the union of Middle + Distal, whereas “distal” refers exclusively to the most 3’ PAS.

8. What is the row labeled “ALL” in Figure 2B referred to? There is no clear explanation in the content (line 252).

Response: We thank the reviewer for pointing out the lack of clarity. In Figure 2B, the row labeled “ALL” represents the cell type proportions calculated across all samples combined (non-viral control and COVID-19 groups together), rather than within each group separately. This was intended to provide an overview of the overall cellular composition across the dataset. We have updated the legend accordingly.

9. Please provide the statistical values run for Supplementary Figure 3 as KS test has been performed.

Response: We thank the reviewer for the suggestion. The statistical values from the Kolmogorov-Smirnov (KS) tests have already been provided in the legend of Supplementary Figure 3, where the corresponding P values are reported for each comparison. To make this clearer, we have further emphasized in the legend that the values shown are derived directly from the KS test.

10. Line 263: please clearly discriminate the difference between non-proximal PAS and distal PAS.

Response: We thank the reviewer for the comment. We have now clarified the distinction between “non-proximal” and “distal” PAS in the Materials and Methods section (lines 158-160 in the revised manuscript).

11. In Materials and Methods, please detail the criteria for the selection of up-regulated and down-regulated PAS (Figure 2F). Are they defined based on sc-RNA-seq, or did the authors perform RNA-seq in this work as well (although I did not see it mentioned)?

Response: We appreciate the reviewer’s comment. The up-regulated and down-regulated PAS in Figure 2F were defined based on single-cell APA analysis, not bulk RNA-seq. Specifically, for each cell type, we used the FindMarkers function in Seurat to compare PAS usage between COVID-19 cases and non-viral controls within that cell type. Significantly altered PAS were defined as those with an adjusted p-value < 0.05 and an absolute log2 fold change > 0.25. PAS with log2FC > 0.25 were classified as up-regulated, and those with log2FC < -0.25 as down-regulated. We have now added the definition to the Materials and Methods section (lines 168-174 in the revised manuscript).

12. It is recommended that the authors should perform normalization of the number of PAS plotted in Figure 2F and perform a statistical test across cell types.

Response: We thank the reviewer for the suggestion. While per-cell CPM normalization was applied before differential PAS detection, we agree that the number of significant PAS per cell type in Figure 2F may be influenced by the total number of testable PAS. To address this, we calculated, for each cell type, the proportion of significant PAS (number of significant PAS / number of testable PAS, separately for up- and down-regulated PAS) and performed statistical comparisons across cell types for each PAS category using contingency-table tests (Chi-squared test, or Fisher’s exact test when expected counts were small; Benjamini-Hochberg adjusted). These normalized proportions and p-values are provided in Supplementary Table 10.2. As the trends and conclusions remain unchanged, we retained the original bar plot in Figure 2F and added a note in the legend to refer to the supplementary table.

13. Line 304: Once again, please clearly elaborate on how the PAS expression of cell types is computed here.

Response: We appreciate the reviewer’s comment. We have now elaborated in the Materials and Methods section (lines 168-174 in the revised manuscript) how PAS expression for each cell type was computed. Specifically, cell-type PAS expression was derived from the SCAPTURE PAS-by-cell UMI matrix after per-cell CPM normalization. For each annotated cell type (Idents in Seurat), we calculated the mean CPM per PAS across cells (AverageExpression). Group comparisons (COVID-19 vs. control within the same cell type) were performed on per-cell CPM using FindMarkers (Wilcoxon rank-sum test) to obtain average log₂ fold-change and adjusted P values, with significance defined as adjusted P < 0.05 and |avg log₂FC| > 0.25.

14. The content between lines 336-343 should move to materials and methods with a clear explanation about the methodology used for the correlation between the PAS site usage and gene expression.

Response: We thank the reviewer for the constructive suggestion. The content between lines 336-343 has been moved to the Materials and Methods section, where we now provide a clearer and more detailed explanation of the methodology. Specifically, we analyzed glutamatergic neurons and GABAergic interneurons to investigate the correlation between APA usage at 3’-UTR and gene expression. PAS located in the 3’-UTR were extracted and analyzed using movAPA to calculate usage differences between the COVID-19 and control groups. The difference in usage was defined as the usage value in the infected group minus that in the control group, with an absolute value greater than 0.1 considered a significant APA usage change. These results were then integrated with gene expression changes to classify genes into four categories corresponding to the quadrants in Figures 3A and 3B.

15. How many significant genes are present in each quadrant? Do those genes possess similar or distinct functional annotations?

Response: We thank the reviewer for the question. Supplementary Table 6 now includes both the list of all 408 significant genes across cell types and the number of significant genes in each quadrant. Functional annotation enrichment analysis for genes in each quadrant is shown in Figure 4F, Supplementary Figure 6, and Supplementary Table 8. As described in the Results section (lines 468-481), genes in different quadrants display distinct functional profiles. For example, terms related to neuronal processes, including neuron projection development and regulation of neurotransmitter levels, were enriched in quadrant 4 for glutamatergic neurons, GABAergic interneurons, and oligodendrocytes. Long-term depression and GnRH secretion were enriched in both glutamatergic neurons and GABAergic interneurons, whereas trans-synaptic signaling and regulation of system process were enriched in astrocytes and oligodendrocytes in quadrant 2. In contrast, quadrant 1 was enriched for RNA splicing, glial cell differentiation, and Parkinson disease. These results indicate that genes from different quadrants exhibit distinct functional annotations.

16. In Figure 3C, oligodendrocytes showed an obvious decrease in NEFL expression as well. Why is this cell type not mentioned?

Response: We thank the reviewer for the comment. In Figure 3C, the UMAP visualization shows a decrease in NEFL expression in oligodendrocytes in the COVID-19 group. However, NEFL expression in this cell type is relatively low, as indicated by the narrow width of the violin plots in Figures 3D and 3E, which reflect the distribution density of expression values. In contrast, glutamatergic neurons and GABAergic interneurons exhibit much higher NEFL expression levels, with a clear and statistically significant reduction in the COVID-19 group (Figure 3D) and decreased PAS usage at the NEFL-3 site (Figure 3E). For this reason, our discussion focused on these two major expressing cell types.

17. Line 370: A few sentences for the explanation of NEFL-3 will aid the readers to better understand the message that the authors attempted to deliver between the lines. I cannot follow why the authors suddenly mentioned NEFL-3. Is it a subfamily under NEFL?

Response: We thank the reviewer for pointing out the need for clarification. NEFL-3 does not represent a subfamily of NEFL, rather, it refers to a PAS of the NEFL transcript, as annotated in our APA analysis. In Figure 3E, we present the PAS usage results, where “NEFL-3” indicates the third annotated PAS site for NEFL. The observed decrease in NEFL-3 usage after infection (Figure 3E) suggests that COVID-19 may affect NEFL transcript processing at the 3’-UTR level, potentially contributing to the altered NEFL expression shown in Figures 3C and 3D. We have revised the text in Line 429 to clarify this point for readers.

18. In the section from line 388, the authors attempted to test the prediction power of the APA usage to miRNAs; however, the results shown here are only a correlation, instead of a prediction. The authors can run a simple logistic regression to verify their hypothesis.

Response: We thank the reviewer for the comment. We would like to clarify that our analysis does not aim to use APA events to predict the existence of miRNAs. Rather, we investigate how APA changes in infection samples could potentially alter miRNA binding by adding or removing miRNA target sites in the 3’UTR. To achieve this, we applied three established databases-miRcords, miRTarBase, and TarBase-to the genes affected by APA changes and identified 2,404 miRNAs whose target sites are predicted to be impacted (Supplementary Table 7). Thus, our results represent a correlation between APA changes and potential miRNA target site alterations, not a direct predictive model of miRNA occurrence. Regarding Figures 4A and 4B, we have revised the Results section to explicitly describe them. These figures summarize the numbers of miRNA target sites gained or lost in each major cell type as a result of APA changes observed in infection samples.

19. Line 441: please explain the potential functions of the rest 141 genes that are also retrieved from 4 quadrants but are not assigned to disease risk genes.

Response: We thank the reviewer for the comment. In addition to disease-risk genes, we identified 141 genes from the four quadrants that were not assigned to known disease-associated gene sets. These genes are listed in Supplementary Table 10.3. We further performed GO enrichment analysis for these genes, and the results are presented in Supplementary Table 10.4. The enriched GO terms suggest that these genes are involved in multiple fundamental biological processes, including: Quadrant 1: Predominantly RNA‐related processes such as mRNA splicing, processing, and metabolism, as well as regulation of microtubule‐based processes and ion transport. Quadrant 2: Enriched for cellular structural organization (e.g., cilium and cell projection assembly, endomembrane system organization), protein degradation, viral processes, and response to ER stress. Quadrant 3: Mainly related to regulation of transcription factor activity and mitotic cell cycle transitions. Quadrant 4: Associated with translation and cognitive processes (learning and memory). These results indicate that although these genes are not linked to known brain disease risk in current databases, they participate in essential molecular and cellular functions that could be relevant to cellular responses and pathophysiological processes during infection.

Discussion

In this work, the authors attempt to present the concept that APA could be used as a potential biomarker for post-COVID patients. I could agree that here the authors demonstrated a correlation between different usage of PAS coupled with host transcriptomics before and after SARS-CoV-2 infection. However, I see weak support for results that are robust enough for the authors to make this conclusion. Especially, as previously mentioned, no prediction models were tested in this study. Furthermore, it is not clear to me how APA can also serve as a potential therapeutic target. Do the authors aim to target viruses or cure neurological diseases?

Response: We thank the reviewer for this important comment. We agree that our current data provide correlative evidence of APA changes in neural cells after SARS-CoV-2 infection but do not establish predictive models or causal links. To address this, we have revised the Discussion to tone down our statements and to clarify that APA should be considered a potential rather than definitive biomarker. We now emphasize that this study provides a foundation for future work in which prediction models or prospective patient cohorts could be tested. Regarding the therapeutic implications, our intention was not to suggest that APA itself is directly targetable to eliminate SARS-CoV-2. Instead, we clarified that APA changes may reveal dysregulated molecular pathways (e.g., immune signaling, neuronal function) that could in turn highlight therapeutic entry points for neurological complications of COVID-19. We revised the text to explicitly state this scope and to avoid any confusion with direct antiviral strategies.

Minor concerns:

1. Line 190: Please cite Supplementary Fig. 1C for the description of cell markers corresponding to different subsets of cell types.

Response: We have added the citation to Supplementary Fig. 1C in Line 228 to indicate the corresponding violin plots showing expression levels of cell-type-specific markers.

2. In Supplementary Fig. 1D, I recommend replacing the column annotation “cluster” with “cell types” or “cell subsets” because no clustering was performed across cell types, and it was confusing to call it “cluster”.

Response: We thank the reviewer for pointing this out. In Supplementary Fig. 1D, the column annotation “cluster” has been changed to “cell types” for clarity.

**Reviewer #4:**

Major comments

1) Figures 2A and 2C: The visual differences between groups are subtle and not readily apparent by eye. In Figure 2A, the zoomed-in view may exaggerate the difference. Please provide the exact numerical differences and clarify whether the observed changes are statistically significant.

Response: We thank the reviewer for this important point. Figures 2A and 2C are UMAP-based visualizations intended to illustrate the global pattern of APA usage across cell types and groups. We fully agree that visual inspection alone may not convey statistically significant shifts, and we appreciate the opportunity to clarify this. To quantitatively assess APA changes, we performed statistical analysis of Proximal Usage Index (PUI) distributions in each cell type using Kolmogorov-Smirnov (K-S) tests, as detailed in Supplementary Figure 3 and the revised Methods. Specifically, the cumulative distribution plots in Supplementary Figure 3A-G show side-by-side comparisons of scaled PUI values between the COVID-19 and non-viral control groups for each cell type.

2) Figure 3A: To improve clarity, consider labeling the proximal and distal APA events directly within their corresponding quadrants. This would make the figure easier to interpret.

Response: Thank you for the suggestion. We believe there may have been a misunderstanding regarding the axes in Figure 3A. In this plot, the x-axis represents gene expression changes (avg_logFC between infected and control groups), while the y-axis represents changes in APA usage (infected group minus control group, with |Δusage| > 0.1 considered significant). Positive y-values correspond to a relative shift toward distal PAS usage in the infected group, and negative y-values correspond to a shift toward proximal PAS usage. Because the quadrants are defined by combinations of these two parameters (expression change and APA usage change), labeling them simply as “proximal” or “distal” would be misleading. Instead, we have ensured that the legend and figure caption clearly describe the meaning of the axes and each quadrant, so that the relationship between APA shift direction and expression change is fully transparent to readers.

3) Cell Type Proportions: Including explicit percentages or cell counts for major populations (e.g., “glutamatergic neurons comprise X %”) in the main text—not solely in Supplementary Figure 1A—would enhance the clarity and accessibility of the results.

Response: We thank the reviewer for this helpful suggestion. In the revised manuscript, we have added explicit percentages of each major cell type in the control group to the main text. This addition improves clarity and accessibility of the results.

4) Figure 3F: The visual differences are not apparent by eye. Please include the exact values or fold-changes to better support the interpretation.

Response: We thank the reviewer for this comment. Figure 3F was designed as a qualitative illustration of PAS distribution at the NEFL locus using IGV, rather than as a quantitative analysis. As the reviewer correctly notes, the visual differences are subtle. To avoid overinterpretation, we revised the text to explicitly acknowledge that Figure 3F serves only as an example of read coverage and PAS annotation, while the evidence for APA dynamics is mainly supported by the genome-wide analyses in Figures 3A-3B. We therefore describe the NEFL observation more cautiously as a “sample-specific trend toward distal PAS usage”.

5) Sample Metadata and Quality Metrics: For transparency and reproducibility, include key donor/sample metadata (e.g., age, sex, health status), along with technical metrics such as UMI counts per nucleus, read depth, and mapping quality. This will help validate consistency across the seven samples.

Response: We thank the reviewer for this suggestion. Key donor metadata, including age, sex, group, number of nuclei, and COVID-19 neurological symptoms are now summarized in Supplementary Table 10.5 for quick reference. The complete metadata set, including additional parameters such as cause of death, and other technical details, is available in the original publication (Nature, 2021, 595:565–571).

6) Statistical Analysis: Clearly specify the statistical tests used (e.g., Kolmogorov–Smirnov test) and the thresholds for significance when comparing APA shifts between infected and control groups.

Response: We thank the reviewer for pointing this out. We have revised the Method section to explicitly state the tests and thresholds used. Specifically, for differential PAS expression, group comparisons (COVID-19 vs. control within the same cell type) were performed on per-cell CPM using the Wilcoxon rank-sum test implemented in FindMarkers, with significance defined as adjusted P < 0.05 and |avg_log₂FC| > 0.25. For APA usage shifts, cumulative distribution plots of scaled proximal usage index (PUI) values were compared between groups using the Kolmogorov-Smirnov test, with significance defined as P < 0.05.
