# Supplementary material for: Single-cell alternative polyadenylation analysis reveals mechanistic insights of COVID-19-associated neurological and psychiatric effects
Source: PLoS One. 2025 Dec 26;20(12):e0324689. doi: 10.1371/journal.pone.0324689 (PMC12742782; doi:10.1371/journal.pone.0324689)
Supplement: Attachment — Submitted filename: Point by point response2.docx [file pone.0324689.s022.docx]

Dear Editor,

Thank you for the decision letter, informing us that our manuscript is potentially acceptable for publication in *PLOS One*. We would like to thank reviewers and editor for their helpful comments and suggestions. We have carefully addressed their concerns as outlined in the attached file. Point-by-point responses are followed by this cover letter. We hope that we have adequately addressed all reviewers’ and editor’s concerns and have made this revised manuscript acceptable for publication in *PLOS One.*

Sincerely,

Wanshan Ning, PhD

Professor of Bioinformatics,

Institute for Clinical Medical Research

the First Affiliated Hospital of Xiamen University,

Xiamen University

55 Zhenhai Road, Xiamen, Fujian, China 361003

E-mail: ningwanshan@xmu.edu.cn

**Responses to the editor and reviewers**

We thank the editor and reviewer for their thorough assessment and thoughtful suggestions, which helped improve this manuscript. We have incorporated the suggestions in the revised version of this manuscript, and a point-by-point response to the reviewer’s comments is presented below.

**Reviewer #1:** (No Response)

**Reviewer #2:** I appreciate the cautiousness brought to the text in this new version of the manuscript. It is now more scientifically honest on the fact that this paper is purely exploratory. Most English sentences that were problematic were corrected. The only grammatical/syntax remark is the following:

Lines 160-167 (track change version): While the authors have nicely clarified their definition of the PUI parameter, the text in black (track change version) should now be removed as it is a badly written repetition of the above sentences.

Response: We thank the reviewer for this helpful suggestion. We have removed the redundant sentences in lines 160-167 that repeated the description of the PUI definition. The revised paragraph now concisely defines PUI without duplication.

While several Figure corrections were asked, the authors only addressed comments through text adjustments. Also:

- Poly(A) is still a confusing term

Response: We appreciate the reviewer’s insightful comment. To clarify the terminology, we have now explicitly defined “polyadenylation” and “poly(A) site (PAS)” in the Introduction (Lines 70-74).

- Line 222 (track change version): space missing “generating16,020”

Response: We appreciate the reviewer’s careful reading. The missing space in “generating16,020” has been corrected to “generating 16,020” in the revised manuscript.

- Figure 1G: The authors apparently understood my initial comment about the higher detection rate in high abundance populations. Since it is feasible and data are available, the authors should quantify the impact of the cell abundance on the detection sensitivity. If the authors RANDOMLY subset the high abundance cell populations, how does this affect their graph?

Response: We thank the reviewer for this insightful suggestion. To evaluate whether the higher PAS detection rate in abundant populations (e.g., Glutamatergic neurons) is driven by cell abundance, we performed a random subsampling analysis. Specifically, we randomly downsampled Glutamatergic neuron nuclei to 50% and 25% of the original cell number and recalculated the number of detected PASs per gene using the same method applied to the full dataset. During this process, we identified and corrected a minor counting error in the original Figure 1G, which was caused by including non-numeric identifiers in the expression matrix during zero-count calculation. After correction, the overall pattern remained consistent, and the conclusions regarding the relationship between cell abundance and PAS detection rate were unaffected. The revised figure and corresponding values have been updated accordingly in the manuscript. After correction, the results are as follows: 100% (full dataset): 159,026 PAS, 50% subsampling: 155,084 PAS, and 25% subsampling: 148,749 PAS. These results indicate that the total number of detected PAS decreases only slightly with cell number reduction, suggesting that the observed higher detection rate in abundant cell populations is not primarily driven by cell abundance but rather reflects genuine biological variation in PAS diversity.

- Figure 2E-F: While I now understand the authors with the corrections brought, I’d like to obtain a visualisation of the Slope Charts (or heatmaps with Log2FC as scale instead of barplots) I suggested in the first round of review (it should have been included in the Rebuttal Letter instead of stating obscurely “less effective to convey the information”). I wanted to obtain it by myself but don't have access to the table “./sang_tot_1201.csv” on the GitHub page and the link provided by the authors requires an account. Data should be accessible on public repositories without any restriction. The point of this comment is to obtain a visualisation of all the up/down-regulations to have a better idea of what is a up/down-regulated PAS. The suggestions do not have to be included in the manuscript but would strengthen the confidence of the reviewers (myself) in the proposed manuscript.

Response: We sincerely thank the reviewer for the constructive follow-up comment and the opportunity to clarify this point further. Following the reviewer’s suggestion, we have now generated a heatmap showing the log2FC of significantly regulated PAS (p < 0.05) across all major brain cell types, separately for distal and proximal PAS (figure provided below). This visualization provides a concise overview of global up- and down-regulation trends among PAS sites with statistically significant changes. Each row represents an individual PAS, and each column represents a specific cell type-PAS subtype combination (e.g., Neuron_Distal, Astrocyte_Proximal). Red indicates increased PAS usage, while blue indicates decreased usage. Regarding data access, we apologize for the inconvenience caused by the GitHub repository restriction. The file “sang_tot_1201.csv” has now been made publicly available without login requirements on an unrestricted repository [https://github.com/yinggu94/APA], ensuring full accessibility and reproducibility. We hope that this additional visualization and open data access address the reviewer’s concern and strengthen confidence in the robustness of our analyses.

- Lines 393 - 402 (track change version): I’m ok with the authors response. They should use the word “meaningful” in their manuscript instead of “significant” if that’s what they meant from the beginning. They should also cite the reference they provided to support their statement.

Response: We thank the reviewer for this helpful suggestion. In the revised manuscript, we have replaced the word “significant” with “meaningful” to better convey our intended interpretation. Additionally, we have added the corresponding reference we previously mentioned to support this statement.

**Reviewer #3:** I thank the authors for their efforts in answering all my questions. In this revised version of the manuscript, the authors have provided enough information on methods utilized in this work, reinforced the statistical test wherever applicable, and clarified the issues arising in the previous version of the manuscript. At this stage, I do not have further comments on this version of the manuscript.

**Reviewer #4:** The authors have thoroughly addressed the major concerns raised during the previous review. The revised manuscript shows improved clarity, methodological transparency, and appropriate caution in interpreting qualitative data. The only remaining recommendation is to consider adding a compact summary of technical metrics (e.g., UMI counts, sequencing depth, mapping quality) across samples to further support reproducibility.

Once this minor point is addressed, I believe the manuscript will be suitable for publication.

Response: We appreciate the reviewer’s positive feedback and helpful suggestion. We have now added a compact summary of technical metrics, including the median/mean UMI per nucleus and median/mean genes per nucleus for each sample, which are presented in Supplementary table 10.7 of the revised manuscript. Unfortunately, sequencing depth and mapping quality were not reported in the original publication from which the dataset was obtained, and therefore could not be included in this summary.
